# Supplementary material for: mRNA vaccines encoding fusion proteins of monkeypox virus antigens protect mice from vaccinia virus challenge
Source: Nat Commun. 2023 Sep 22;14:5925. doi: 10.1038/s41467-023-41628-5 (PMC10516993; doi:10.1038/s41467-023-41628-5)
Supplement: Supplementary file 1 — Supplementary Information [file 41467_2023_41628_MOESM1_ESM.pdf]

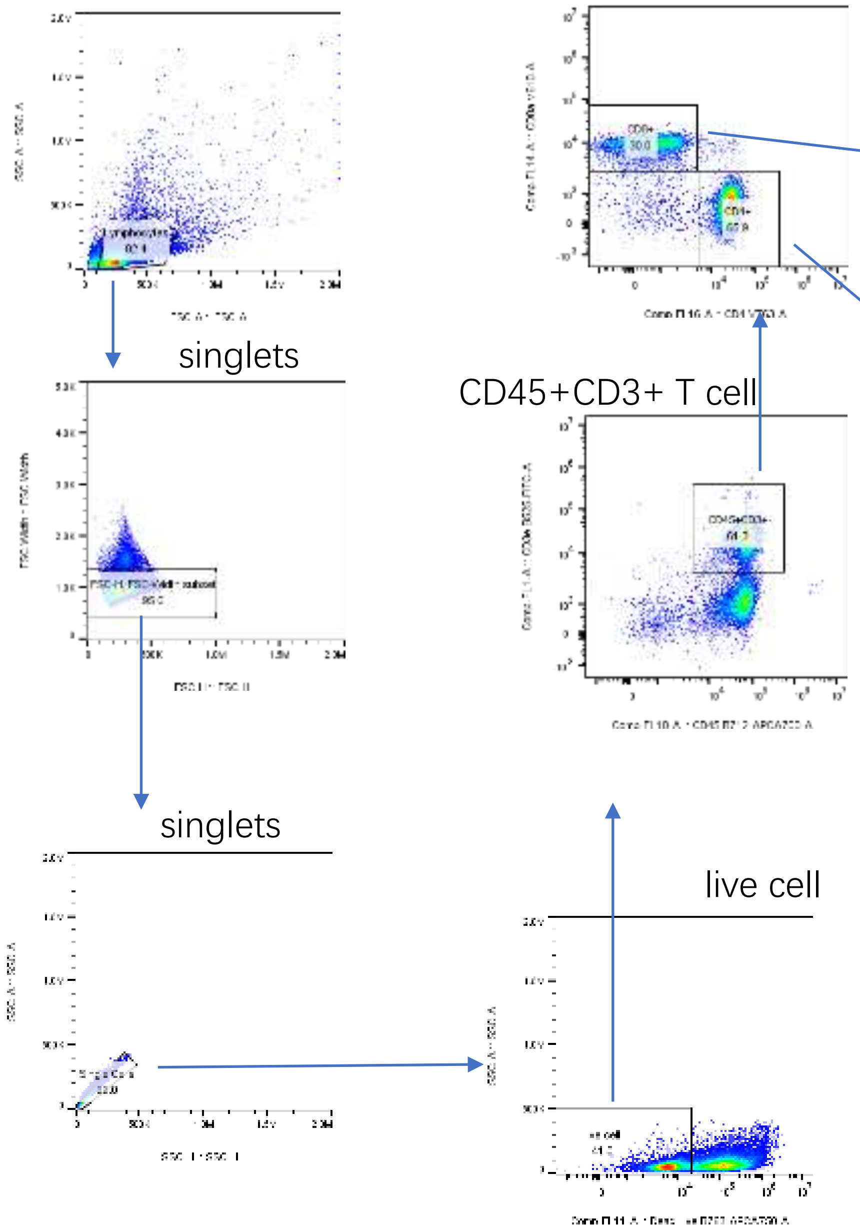


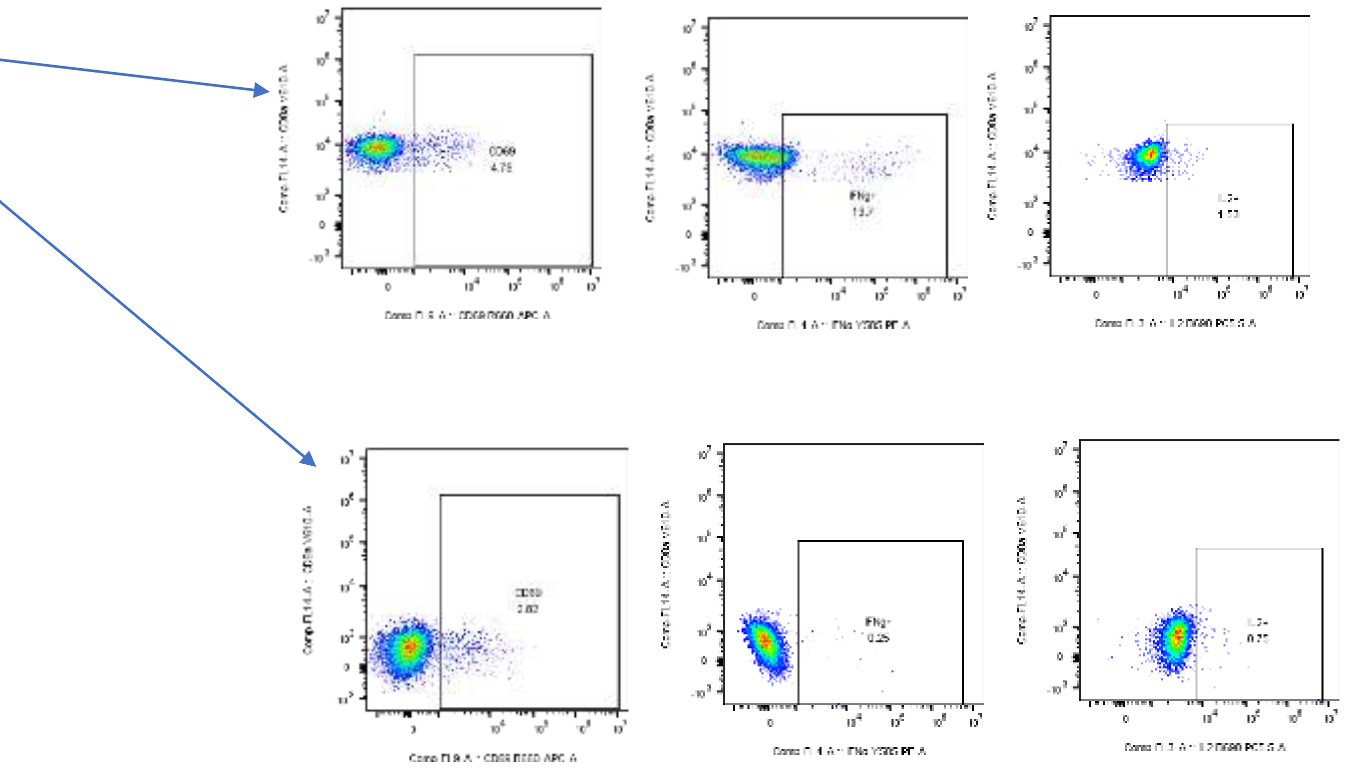

Supplementary Figure 1. Gating strategy in flow cytometric analysis.

Firstly, duplets and dead cells were excluded. Then CD45+CD3e+CD8-CD4+ T cells or CD45+CD3e+CD4-CD8+ were gated. After that, CD69+, IFNγ+ or IL2 single positive cells were gated in either CD4 T cell or CD8 T cell according to the isotype controls.
