# Supplementary material for: mRNA vaccines encoding fusion proteins of monkeypox virus antigens protect mice from vaccinia virus challenge
Source: Nat Commun. 2023 Sep 22;14:5925. doi: 10.1038/s41467-023-41628-5 (PMC10516993; doi:10.1038/s41467-023-41628-5)
Supplement: Supplementary file 3 — Reporting Summary [file 41467_2023_41628_MOESM3_ESM.pdf]

nature portfolio

Corresponding author(s): Xiaoming Yang, William Jia

Last updated by author(s): Aug 18, 2023

Reporting Summary

Nature Portfolio wishes to improve the reproducibility of the work that we publish. This form provides structure for consistency and transparency in reporting. For further information on Nature Portfolio policies, see our Editorial Policies and the Editorial Policy Checklist.

Statistics

For all statistical analyses, confirm that the following items are present in the figure legend, table legend, main text, or Methods section.

n/a	Confirmed	
☐	☒	The exact sample size (*n*) for each experimental group/condition, given as a discrete number and unit of measurement
☐	☒	A statement on whether measurements were taken from distinct samples or whether the same sample was measured repeatedly
☐	☒	The statistical test(s) used AND whether they are one- or two-sided<br>*Only common tests should be described solely by name; describe more complex techniques in the Methods section.*
☒	☐	A description of all covariates tested
☐	☒	A description of any assumptions or corrections, such as tests of normality and adjustment for multiple comparisons
☐	☒	A full description of the statistical parameters including central tendency (e.g. means) or other basic estimates (e.g. regression coefficient) AND variation (e.g. standard deviation) or associated estimates of uncertainty (e.g. confidence intervals)
☐	☒	For null hypothesis testing, the test statistic (e.g. *F*, *t*, *r*) with confidence intervals, effect sizes, degrees of freedom and *P* value noted<br>*Give P values as exact values whenever suitable.*
☒	☐	For Bayesian analysis, information on the choice of priors and Markov chain Monte Carlo settings
☒	☐	For hierarchical and complex designs, identification of the appropriate level for tests and full reporting of outcomes
☒	☐	Estimates of effect sizes (e.g. Cohen's *d*, Pearson's *r*), indicating how they were calculated

Our web collection on statistics for biologists contains articles on many of the points above.

Software and code

Policy information about availability of computer code

Data collection	Peiqing JS-M6P ECL SensiCapture; FlowJo V.10.8.1;
Data analysis	Microsoft Office Excel 2010; GraphPad Prism 9; Microsoft Office Excel 2010;

For manuscripts utilizing custom algorithms or software that are central to the research but not yet described in published literature, software must be made available to editors and reviewers. We strongly encourage code deposition in a community repository (e.g. GitHub). See the Nature Portfolio guidelines for submitting code & software for further information.

Data

Policy information about availability of data

All manuscripts must include a data availability statement. This statement should provide the following information, where applicable:

- Accession codes, unique identifiers, or web links for publicly available datasets
- A description of any restrictions on data availability
- For clinical datasets or third party data, please ensure that the statement adheres to our policy

Source data are provided with this paper.

Human research participants

Policy information about studies involving human research participants and Sex and Gender in Research.

Reporting on sex and gender	This study is not involved in human research.
Population characteristics	This study is not involved in human research.
Recruitment	This study is not involved in human research.
Ethics oversight	This study is not involved in human research.

Note that full information on the approval of the study protocol must also be provided in the manuscript.

Field-specific reporting

Please select the one below that is the best fit for your research. If you are not sure, read the appropriate sections before making your selection.

☒ Life sciences ☐ Behavioural & social sciences ☐ Ecological, evolutionary & environmental sciences

For a reference copy of the document with all sections, see nature.com/documents/nr-reporting-summary-flat.pdf

Life sciences study design

All studies must disclose on these points even when the disclosure is negative.

Sample size	according to little difference between individuals in same treatment in preliminary experiment, at least four mice were chosen in each group, ranging from 4 to 20.
Data exclusions	In the ELISA assay, some samples were randomly selected and used in preliminary experiment and their data not contained in final experiments.
Replication	the blots results were repeated at least 3 times. some animal experiments were repeated twice and some repeated once.
Randomization	Different groups of mice were randomly feed in cages with same conditions and treated by same methods. Samples from cells or mice were collected at the same time point and examined with same methods.
Blinding	Investigators were blinded for data collection but not for data analysis because we need to analyze which group behave better and why.

Reporting for specific materials, systems and methods

We require information from authors about some types of materials, experimental systems and methods used in many studies. Here, indicate whether each material, system or method listed is relevant to your study. If you are not sure if a list item applies to your research, read the appropriate section before selecting a response.

Materials & experimental systems

n/a	Involved in the study	
☐	☒	Antibodies
☐	☒	Eukaryotic cell lines
☒	☐	Palaeontology and archaeology
☐	☒	Animals and other organisms
☒	☐	Clinical data
☒	☐	Dual use research of concern

Methods

n/a	Involved in the study	
☒	☐	ChIP-seq
☐	☒	Flow cytometry
☒	☐	MRI-based neuroimaging

Antibodies

Antibodies used	M1R Human Mab (OkayBio, R403k5), 1:1000 A35R Mouse Mab (Sino Biological, 40886-M0017), 1:1000 GAPDH Mouse Mab (Sangon, D190090), 1:2000. HRP-goat anti human IgG (Sangon, D110150), 1:5000; HRP-goat anti mouse IgG (Sangon, D110087), 1:5000.
Validation	the manufactures state that their antibodies are verified for the relevant applications.

Eukaryotic cell lines

Policy information about cell lines and Sex and Gender in Research

Cell line source(s)	Vero and 293T cell lines were bought from ATCC.
Authentication	None of the cell lines used were authenticated because we assume ATCC has done this work. Besides, we frozen the cell lines in aliquots upon its arrival.
Mycoplasma contamination	All cell lines tested negative for mycoplasma contamination.
Commonly misidentified lines (See ICLAC register)	None

Animals and other research organisms

Policy information about studies involving animals; ARRIVE guidelines recommended for reporting animal research, and Sex and Gender in Research

Laboratory animals	Balb/c mice, female, 7-8 week old.
Wild animals	No wild animals were used.
Reporting on sex	Female mice were used in this study because female mice are more gentle than male mice. Besides, androgen is largely variant in male mice and it can affect immune response.
Field-collected samples	The study did not involve samples collected from the field.
Ethics oversight	Mouse housing and experimental procedures were approved by Animal Research Committee of Virogin in accordance with national guidelines.

Note that full information on the approval of the study protocol must also be provided in the manuscript.

Flow Cytometry

Plots

Confirm that:

☒ The axis labels state the marker and fluorochrome used (e.g. CD4-FITC).

☒ The axis scales are clearly visible. Include numbers along axes only for bottom left plot of group (a 'group' is an analysis of identical markers).

☒ All plots are contour plots with outliers or pseudocolor plots.

☒ A numerical value for number of cells or percentage (with statistics) is provided.

Methodology

Sample preparation	Spleens were mechanically disrupted and treated with ACK lysis buffer for red blood cell removal. Then spleen cells were filtered through a 70 µm cell strainer and resuspended in RPMI1640 medium.
Instrument	Cytoflex flow cytometer (Beckman)
Software	FlowJo V.10.8.1
Cell population abundance	Live cells accounted for 35-45 percentages for total singlets. CD3e+ T cells accounted for 50-60% of total CD45+ live cells. CD4 T cells accounted for 65-70% of total CD3e+ T cells, and CD8 T cells accounted for 25-35% of total CD3e+ T cells.
Gating strategy	Firstly, duplets and dead cells were excluded. Then CD45+CD3e+CD8-CD4+ T cells or CD45+CD3e+CD4-CD8+ were gated. After that, CD69+, IFNγ+ or IL2 single positive cells were gated in either CD4 T cell or CD8 T cell according to the isotype controls.

☒ Tick this box to confirm that a figure exemplifying the gating strategy is provided in the Supplementary Information.
